# Supplementary material for: Encourage or inhibit: A study on the impact of corporate digital transformation on management’s tone manipulation of information disclosure
Source: PLoS One. 2025 Feb 25;20(2):e0317503. doi: 10.1371/journal.pone.0317503 (PMC11856518; doi:10.1371/journal.pone.0317503)
Supplement: S1 File — (DOCX) [file pone.0317503.s001.docx]

Appendix 1: Keywords of Enterprise Digital Transformation

| **Category** | **Keywords** |
| --- | --- |
| Artificial Intelligence | Artificial Intelligence (AI), Business Intelligence (BI), Image Understanding, Investment Decision Support System, Intelligent Data Analysis, Intelligent Robot, Machine Learning (ML), Deep Learning (DL), Semantic Search, Biometric Technology, Facial Recognition, Speech Recognition, Identity Verification, Autonomous Driving, Natural Language Processing (NLP). |
| Blockchain | Blockchain, Digital Currency, Distributed Computing, Differential Privacy Technology, Smart Financial Contract |
| Big Data Technology | Big Data, Data Mining, Text Mining, Data Visualization, Heterogeneous Data, Credit Reporting, Augmented Reality (AR), Mixed Reality (MR), Virtual Reality (VR). |
| Cloud Computing | Cloud Computing, Stream Computing, Graph Computing, In-Memory Computing, Secure Multi-Party Computation (SMC), Neuromorphic Computing, Green Computing, Cognitive Computing, Converged Architecture, Hundred-Million-Level Concurrency, Exabyte-Level Storage (EB-Level Storage), Internet of Things (IoT). |
| Digital Technology  Applications | Mobile Internet, Industrial Internet, Mobile Connectivity, Internet Healthcare, E-commerce, Mobile Payment, Third-Party Payment, Near Field Communication Payment (NFC Payment), Smart Energy, Business-to-Business (B2B), Business-to-Consumer (B2C), Consumer-to-Business (C2B), Consumer-to-Consumer (C2C), Online-to-Offline (O2O), Online Connection, Smart Wearables, Smart Agriculture, Intelligent Transportation, Smart Healthcare, Intelligent Customer Service, Smart Home, Robo-Advisory, Smart Culture and Tourism, Smart Environmental Protection, Smart Grid, Intelligent Marketing, Digital Marketing, Unmanned Retail, Internet Finance, Digital Finance, Financial Technology (Fintech), Quantitative Finance, Open Banking |
