# Supplementary material for: Encourage or inhibit: A study on the impact of corporate digital transformation on management’s tone manipulation of information disclosure
Source: PLoS One. 2025 Feb 25;20(2):e0317503. doi: 10.1371/journal.pone.0317503 (PMC11856518; doi:10.1371/journal.pone.0317503)
Supplement: Attachment — Submitted filename: Response to Reviewers - revision2.docx [file pone.0317503.s004.docx]

**Response to reviewers**

Dear Reviewers：

We are very grateful for your valuable review comments on our study " Encourage or inhibit: A study on the impact of corporate digital transformation on management's tone manipulation of information disclosure", which has significant guidance for the revision and improvement of our study and the enhancement of academic research level!

After carefully reading and thinking about the review comments, we have made the following revisions. Below, the authors will reply to the modification suggestions raised by the reviewers one by one.

***（The blue font part is the reviewer's comments. The black bold font indicates the author's modification explanation, and the regular black font represents the modified content.）***

# Academic Editor:

(1) Please review your reference list to ensure that it is complete and correct.

**Nine references are added in this minor revision. We review reference list and ensure that it is complete and correct.**

# Reviewer #2:

1. Words, language and style

Run-on sentences are a recurrent issue: the paper contains many overly long sentences with multiple clauses that hinder comprehension.

①This is an example: "Innovation, being the core of digital transformation, means that risk information disclosure often involves product research and development, technological innovation, and other related information, although enhancing the redundancy of risk disclosure can increase firms' information transparency and reduce firms' agency costs..."

Suggestion: Split into two sentences for clarity.

②Here another example: "Managers may exploit this by overstating the benefits and downplaying the challenges of digital transformation through tone manipulation, aiming to elicit market reactions that benefit their stock prices."

Suggestion: Break into two shorter, precise sentences.

③This typo of phrases: "Digital transformation positively affects various aspects of enterprises, including production costs, business performance, competitive advantage, and innovation" are repeated in slightly different forms.

Suggestion: Eliminate redundancy and simplify phrasing.

④Inconsistent Vocabulary: Terms like "media optimism" and "media slant" are used interchangeably, potentially confusing readers.

Suggestion: Standardize terminology throughout the text.

**Thanks for your valuable and constructive suggestions. Your suggestions greatly help me to make the introduction more intuitive and logical. In response to this review comment:**

① This example sentence includes content deleted in the first round of revisions. In the previous round of "Revised Manuscript with Track Changes," all changes were retained. This maybe make the content messy and possibly confusing for reviewers. In this revision, the "Revised Manuscript with Track Changes" file highlights changes in red. This makes it easier for reviewers to read. Meanwhile, we read through the entire paper. We revised long sentences that were overly lengthy or complex.

For example, "This integration, facilitated by digital tools and platforms, is transforming products, processes, services, and organizational structures. " is changed to "Digital tools and platforms facilitate this integration. It is transforming products, processes, services, and organizational structures. "; "Impression management refers to the process where people are often concerned, both consciously and unconsciously, about how others evaluate them, and they subconsciously desire to be viewed positively. " is changed to "Impression management refers to the process where people are often concerned about how others evaluate them, both consciously and unconsciously. They have a subconscious desire to be viewed positively. " All the changes can be seen in the red paragraph.

② We modify this sentence to "Managers may exploit this by overstating the benefits and downplaying the challenges of digital transformation through tone manipulation. They aim to elicit market reactions that benefit their stock prices. "

③ The content of this sentence is repetitive with the previous sentence. We thank the reviewer for pointing this out. We combined this sentence with the previous sentence to reduce redundancy. Meanwhile, we reviewed the entire document. We revised and removed other redundant content. This includes content about the purpose of tone manipulation and the current state of digital transformation risk.

④ We normalized the relevant content to "media slant".

2. One missing references

The key theory like impression management (line 102) lacks foundational references.

Recommendation: Add citations to foundational works (e.g., Goffman).

**Thanks for your valuable and constructive suggestions. In response to this review comment, we added references to related literature based on the existing studies. These include references not only related to impression management. They also include references related to the agency theory, incomplete contract theory, etc.**

·References

[14] Goffman E. Stigma: Notes on the management of spoiled identity. 2009. Simon and schuster.

[15] Leary MR, Kowalski RM. Impression management: A literature review and two-component model, Psychological bulletin. 1990; 107(1): 34-47.

[16] Gardner WL, Martinko MJ. Impression management in organizations, Journal of management. 1988; 14(2): 321-338.

[39] Eisenhardt KM. Agency theory: An assessment and review. Academy of management review. 1989; 14(1): 57-74. https://doi.org/10.5465/amr.1989.4279003

[40] Christensen HB, Nikolaev VV, Wittenberg‐Moerman R. Accounting information in financial contracting: The incomplete contract theory perspective. Journal of accounting research. 2016; 54(2): 397-435. <https://doi.org/10.1111/1475-679X.12108>

[58] Glavas C, Mathews S, Russell-Bennett R. Knowledge acquisition via internet-enabled platforms: Examining incrementally and non-incrementally internationalizing SMEs. International Marketing Review. 2019; 36(1): 74-107. https://doi.org/10.1108/IMR-02-2017-0041

[74] Zhang H. Research on the formation mechanism and influencing factors of internet users' information cocoon in Chinese. Journal of Intelligence. 2021; (10): 166-170,185.

https://doi.org/10.3969/j.issn.1002-1965.2021.10.023

[75] Karlsson N, Loewenstein G, Seppi D. The ostrich effect: Selective attention to information. Journal of Risk and uncertainty. 2009; 38: 95-115.

https://doi.org/10.1007/s11166-009-9060-6

[109] Frank MZ, Goyal VK. Testing the pecking order theory of capital structure. Journal of financial economics. 2003; 67(2): 217-248. https://doi.org/10.1016/S0304-405X(02)00252-0

3. Empirical Analysis

Clarity:While the methodology is comprehensive, the presentation is overly technical in some sections. For instance, the explanation of "Abtone" (line 474) could be simplified for clarity.

Suggestion: Use clear transitions between statistical details and their implications to improve readability.

**Thanks for your valuable and constructive suggestions. In response to this review comment, we revised the variable definition section. This makes the content more logical and easier for readers to understand.**

(1) Digital transformation of enterprises (Digital)

Referring to Qi et al. (2020) [92], this study's main regression analysis quantifies enterprise digital transformation by examining the proportion of digital assets in listed companies. Specifically, the notes to the financial statements in the annual report of the enterprise disclose the details of the intangible assets. We identify keywords related to digital technology, such as "software", "network", "client", "management system" and "smart platform". These items are classified as "digital intangible assets". We add up the number of digital technology intangibles for each company in each year. Then, we calculate the proportion of these intangibles relative to the company's total intangibles for the current year. This proportion is the proxy variable of enterprise digital transformation.

Additionally, we refer to Wu et al. (2021) [93]. We use the frequency of digital transformation keywords in the annual reports of listed companies. This frequency serves as a proxy measure of enterprise digital transformation during robustness testing. Specifically, through text analysis, we identify feature words from five categories: artificial intelligence, blockchain, cloud computing, big data, and digital technology applications (Details in Appendix 1). Using Python 3.7.0, we extract keywords from each company's annual report and count the frequency of these keyword occurrences per company each year. Finally, we calculate the logarithm of the frequency plus one, which serves as a proxy indicator for the enterprise digital transformation.

(2) MD&A tone manipulation (Abtone)

To isolate unusually positive tones, we follow the methodology of Li (2010) [32], Huang et al. (2014) [29]. First, we select the control variables related to Tone to construct the model. Then, we use the residual method to divide Tone into two parts. One part is the normal tone. It reflects the company's fundamentals. The other part is the Abtone. It represents an unusually positive tone that deviates from the company's actual performance level. That is, the residual term from the regression captures the aspect of the text tone that surpasses the company's fundamental realities. It reflects the exaggerated emotional tendencies in management's expressions. This is used as a proxy for management's tone manipulation.

·References

[29] Huang X, Teoh SH, Zhang Y. Tone management. The accounting review. 2014; 89(3): 1083-1113. https://doi.org/10.2308/accr-50684

[32] Li F. The information content of forward‐looking statements in corporate filings—A naïve Bayesian machine learning approach. Journal of accounting research. 2010; 48(5): 1049-1102. <https://doi.org/10.1111/j.1475-679X.2010.00382.x>

[92] Qi HJ, Cao XQ, Liu YX. The influence of digital economy on corporate governance: Analyzed from information asymmetry and irrational behavior perspective in Chinese. Reform. 2020; (04): 50-64.

[93] Wu F, Hu HZ, Lin HY, Ren XY. Enterprise Digital Transformation and Capital Market Performance: Empirical Evidence from Stock Liquidity in Chinese. Management World. 2021; 37(07): 130-144+10. https://doi.org/10.19744/j.cnki.11-1235/f.2021.0097

4. Practical Implications

The paper provides meaningful insights for investors and regulators, but these are scattered.

A solution could be to consolidate these into a dedicated "Practical Implications" section.

**Thanks for your valuable and constructive suggestions. The structure of Chapter Six is indeed unclear. In response to this review comment, we added three subheadings in Chapter Six. These additions are based on the research content. They make it easier for readers to understand the main points of this section.**

6.1 Conclusions

6.2 Practical implications

6.3 Limitations and prospects

5. Redundancy

Several points are repeated across sections (e.g., the benefits and risks of digital transformation). This could make the paper appear unnecessarily lengthy.

Please, take into consideration to consolidate repeated points, particularly in the introduction and literature review.

**Thanks for your valuable and constructive suggestions. We indeed had some unnecessary repetitive descriptions in various sections. We thought more detail would be better but it backfired, making the article too verbose. In response to this review comment, we modified unnecessary repetitions in the text. This includes content about the benefits of digital transformation, the purpose of tone manipulation and the current state of digital transformation risk and so on.**
