# Supplementary material for: Encourage or inhibit: A study on the impact of corporate digital transformation on management’s tone manipulation of information disclosure
Source: PLoS One. 2025 Feb 25;20(2):e0317503. doi: 10.1371/journal.pone.0317503 (PMC11856518; doi:10.1371/journal.pone.0317503)
Supplement: Attachment — Submitted filename: Response to Reviewers.docx [file pone.0317503.s003.docx]

**Response to reviewers**

Dear Reviewers：

We are very grateful for your valuable review comments on our study " Encourage or inhibit: A study on the impact of corporate digital transformation on management's tone manipulation of information disclosure", which has significant guidance for the revision and improvement of our study and the enhancement of academic research level!

After carefully reading and thinking about the review comments, we have made the following revisions. Below, the authors will reply to the modification suggestions raised by the reviewers one by one.

***（The blue font part is the reviewer's comments. The black bold font indicates the author's modification explanation, and the regular black font represents the modified content.）***

# Academic Editor:

(1) Please ensure that your manuscript meets PLOS ONE's style requirements, including those for file naming.

**After reading PLOS ONE style templates, we carefully modify the article format. The major changes are the revision of the title page and the citation and ranking of references. In addition, the uploaded images are also exported through the designated web page, which meets the requirements.**

(2) Please include this amended Role of Funder statement in your cover letter

**We clearly indicate the changes of the financial disclosure in our cover letter.**

(3) Please note that your Data Availability Statement is currently missing the repository name and/or the DOI/accession number of each dataset OR a direct link to access each database.

**Wind, CSMAR and CNRDS databases are used in this paper. Because its data is mainly obtained in a subscription model and is not an open-source database, a specific DOI cannot be provided. We have added database names and website links to the data availability statement. Researchers can access the data by subscribing to the database or by contacting academic institutions with subscription rights. In addition, we uploaded the processed dataset of this paper.**

(4) Please review your reference list to ensure that it is complete and correct.

**We review reference list and ensure that it is complete and correct.**

# Reviewer #1:

(1) Clarity in Variable Definition: While the methodology is sound, some variable definitions could be clearer, particularly for readers who may not be familiar with the context of the Chinese market. It would be beneficial to provide more details on the calculation of variables such as 'Digital' and 'Abtone' in the methodology section.

**Thanks for your constructive suggestions. In response to this review comment, ①we supplement the references and more detailed instructions of digital transformation measurement in 3.2.1, and list the keywords required by the text analysis method in Appendix I; ②we provide more detailed Tone analysis process as well as the computational analysis of the residual (Abtone) in Section 3.2.2.**

3.2.1. Digital transformation of enterprises (Digital)

Referring to Qi et al. (2020) [84], this study's main regression analysis quantifies enterprise digital transformation by examining the proportion of digital assets in listed companies. Specifically, the notes to the financial statements in the annual report of the enterprise disclose the details of the intangible assets. We identify keywords related to digital technology, such as "software", "network", "client", "management system" and "smart platform". These items are classified as "digital intangible assets". We add up the number of digital technology intangibles for each company in each year and then calculate their proportion of the company's intangibles for the current year. This proportion is the proxy variable of enterprise digital transformation.

Additionally, referring to Wu et al. (2021) [85], we adopt the frequency of digital transformation keywords in the annual reports of listed companies as a proxy measure of enterprise digital transformation during the robustness testing. Specifically, through text analysis, we identify feature words from five categories: artificial intelligence, blockchain, cloud computing, big data, and digital technology applications (Details in Appendix 1). Using Python 3.7.0, we extract keywords from each company's annual report and count the frequency of these keyword occurrences per company each year. Finally, we calculate the logarithm of the frequency plus one, which serves as a proxy indicator for the enterprise digital transformation.

3.2.2. Tone manipulation

(1) Calculate MD&A net positive intonation (Tone)

With the development of text analysis technology, scholars built a lexicon with positive and negative emotions in the process of studying intonation. According to the frequency with which these vocabularies appear, a "net positive tone" is constructed to quantify the emotional tendencies of management [26,86]. The Harvard Dictionary's definition of negative words is not completely suitable for the tone analysis requirements in financial reports. To address this gap, Loughran and McDonald (2011) [86] developed a list of negative words suitable for analyzing financial report texts. Therefore, this paper relies on the vocabulary list developed by these two scholars. The study carries out translation through Youdao Dictionary and Jinshan Ciba, as well as retains the Chinese words which most related to the emotion expressed by these English words. And the final list of words included 1076 positive words and 2080 negative words.

There are two methods to calculate net positive intonation. For one thing, Davis and Tama‐Sweet (2012) [32], Huang et al. (2014) [26] defined Tone as the number of positive words minus the number of negative words, and then divided by the total number of words in the annual report. For another, Henry (2008) [22], Price et al. (2012) [87] defined Tone as the number of positive words minus the number of negative words, and then divided by the sum of them. This research chooses the second one to measure the MD&A net positive intonation. A larger Tone value indicates a more positive sentiment in the text.

(2) MD&A tone manipulation (Abtone)

To isolate unusually positive tones, we follow the methodology of Li (2010) [29], Huang et al. (2014) [26], selecting tone model determinants to control for firm fundamentals. The residual method is used to divide the net positive tone into two parts. One part, the normal tone, reflects the company's fundamentals. The other part, known as "tone manipulation," represents an unusually positive tone that deviates from the company's actual performance level. That is, the residual term from the regression captures the aspect of the text tone that surpasses the company's fundamental realities, reflecting the exaggerated emotional tendencies in management's expressions. This is used as a proxy for management's tone manipulation.

·References

[22] Henry E. Are investors influenced by how earnings press releases are written?. The Journal of Business Communication (1973). 2008; 45(4): 363-407.

https://doi.org/10.1177/0021943608319388

[26] Huang X, Teoh SH, Zhang Y. Tone management. The accounting review. 2014; 89(3): 1083-1113. https://doi.org/10.2308/accr-50684

[29] Li F. The information content of forward‐looking statements in corporate filings—A naïve Bayesian machine learning approach. Journal of accounting research. 2010; 48(5): 1049-1102. https://doi.org/10.1111/j.1475-679X.2010.00382.x

[32] Davis AK, Tama‐Sweet I. Managers’ use of language across alternative disclosure outlets: earnings press releases versus MD&A. Contemporary Accounting Research. 2012; 29(3): 804-837. https://doi.org/10.1111/j.1911-3846.2011.01125.x

[84] Qi HJ, Cao XQ, Liu YX. The influence of digital economy on corporate governance: Analyzed from information asymmetry and irrational behavior perspective in Chinese. Reform. 2020; (04): 50-64.

[85] Wu F, Hu HZ, Lin HY, Ren XY. Enterprise Digital Transformation and Capital Market Performance: Empirical Evidence from Stock Liquidity in Chinese. Management World. 2021; 37(07): 130-144+10. https://doi.org/10.19744/j.cnki.11-1235/f.2021.0097

[86] Loughran T, McDonald B. When is a liability not a liability? Textual analysis, dictionaries, and 10‐Ks. The Journal of finance. 2011; 66(1): 35-65.

https://doi.org/10.1111/j.1540-6261.2010.01625.x

[87] Price SM, Doran JS, Peterson DR, Bliss BA. Earnings conference calls and stock returns: The incremental informativeness of textual tone. Journal of Banking & Finance. 2012; 36(4): 992-1011. <https://doi.org/10.1016/j.jbankfin.2011.10.013>

·Appendix 1: Keywords of Enterprise Digital Transformation

| **Category** | **Keywords** |
| --- | --- |
| Artificial Intelligence | Artificial Intelligence (AI), Business Intelligence (BI), Image Understanding, Investment Decision Support System, Intelligent Data Analysis, Intelligent Robot, Machine Learning (ML), Deep Learning (DL), Semantic Search, Biometric Technology, Facial Recognition, Speech Recognition, Identity Verification, Autonomous Driving, Natural Language Processing (NLP). |
| Blockchain | Blockchain, Digital Currency, Distributed Computing, Differential Privacy Technology, Smart Financial Contract |
| Big Data Technology | Big Data, Data Mining, Text Mining, Data Visualization, Heterogeneous Data, Credit Reporting, Augmented Reality (AR), Mixed Reality (MR), Virtual Reality (VR). |
| Cloud Computing | Cloud Computing, Stream Computing, Graph Computing, In-Memory Computing, Secure Multi-Party Computation (SMC), Neuromorphic Computing, Green Computing, Cognitive Computing, Converged Architecture, Hundred-Million-Level Concurrency, Exabyte-Level Storage (EB-Level Storage), Internet of Things (IoT). |
| Digital Technology  Applications | Mobile Internet, Industrial Internet, Mobile Connectivity, Internet Healthcare, E-commerce, Mobile Payment, Third-Party Payment, Near Field Communication Payment (NFC Payment), Smart Energy, Business-to-Business (B2B), Business-to-Consumer (B2C), Consumer-to-Business (C2B), Consumer-to-Consumer (C2C), Online-to-Offline (O2O), Online Connection, Smart Wearables, Smart Agriculture, Intelligent Transportation, Smart Healthcare, Intelligent Customer Service, Smart Home, Robo-Advisory, Smart Culture and Tourism, Smart Environmental Protection, Smart Grid, Intelligent Marketing, Digital Marketing, Unmanned Retail, Internet Finance, Digital Finance, Financial Technology (Fintech), Quantitative Finance, Open Banking |

(2) Generalizability of Findings: The study focuses on Chinese A-share listed companies. Although the sample size is large, the findings may not be directly generalizable to other countries or markets with different regulatory environments or digital transformation stages. Discussing this limitation in more detail would strengthen the study's implications.

**Thanks for your valuable and constructive suggestions. In response to this review comment,** **we add the section Limitations (3) to further clarify the limitations on the generalizability of the research results of the research objects of China's A-share listed companies.**

This study also has several limitations, with future research opportunities in these main areas: ... ... (3) Although this study maintains an adequate sample size, its findings are limited in generalizability as it focuses solely on Chinese A-share listed companies. Countries and markets differ significantly in their information disclosure regulations and stages of digital transformation. Future research should broaden its scope to thoroughly investigate the specific circumstances of various nations concerning information disclosure regulation and digital transformation. Comparative analyses and in-depth investigations are needed on how digital transformation influences managerial strategic information disclosure.

(3) Recent Literature: While the literature review is comprehensive, incorporating more recent studies (post-2021) on digital transformation and tone manipulation could enhance the relevance and impact of the manuscript.

**Thanks for your constructive suggestions. In response to this review comment, we supplement and revise the literature review section. However, there are few studies on the relationship between digital transformation and tone manipulation. So, we further sort out and elucidate the relevant literature in the field of digital transformation and information disclosure. And the corresponding practical examples are given to enhance the section's richness.**

Digital transformation leverages digital technology to redefine corporate strategic thinking, business operations, organizational structures, and business models. It facilitates a comprehensive strategic shift towards data-driven management and innovation [1,48-52]. Research into how digital transformation affects management's tone manipulation is nascent. Nevertheless, existing studies on the impact of digital transformation on information disclosure have showed varied conclusions. From the perspective of information disclosure channels, various platforms facilitate the deep integration of the digital and real economies. Internet platforms enable a positive interactive communication environment, allowing enterprises to use them as vehicles for efficiently transmitting operational information to users. For example, companies can promote and sell products through social media platforms like Weibo, Facebook, and TikTok. Xiaomi represents a successful example of using social media to disrupt traditional startup models. Another example is the interactive platforms launched by the Shenzhen and Shanghai Stock Exchanges, facilitating real-time communication. These platforms provide cost-effective and efficient communication channels, particularly benefiting small and medium investors. This approach significantly enhances the speed and fairness of acquiring information. However, internet platforms may also facilitate management's concealment activities, serving as channels for manipulative information disclosure [30,53,54]. Such strategic disclosure practices can exacerbate market information asymmetry. This form of improper competition lowers the efficiency of information matching [55,56]. From the perspective of information disclosure quality, digital transformation can improve internal control effectiveness by alleviating information asymmetry [57], thus enhances the transparency of internal control information and elevates the level of its disclosure. Externally, it can also improve the accuracy of corporate information disclosure [58,59] or promote herding behavior in corporate ESG information disclosure [60]. However, digital transformation's promotion of green information disclosure might merely reflect superficial compliance by companies [61]. That is, the disclosed information might also include strategic concept speculation behavior [54]. Typically, in the digital economy, companies actively disclose more digital transformation information to signal innovation and competitiveness. However, these disclosures might be more talk than action, merely a tactic of conceptual hype [62,63]. This exaggeration can lead information users to opportunistic behavior, influencing their judgment and decisions. In practice, China's securities regulators have identified instances where misleading disclosures of popular topics confuse investors, leading to stock price manipulation. For example, companies like Bright Oceans Inter-Telecom Corporation and Jiangsu Daybright Intelligent Electric Co., Ltd. were penalized by the CSRC for ambiguously disclosing digital technology applications, such as AI and big data, in reports and interactive platforms. As a result, these companies faced penalties. The companies eventually supplemented their disclosures of related risks under regulatory pressure. Therefore, in the current digitalization era, the extent to which digital transformation improves the capital market's information environment is still under investigation. Consequently, determining the specific impact on corporate information disclosure, particularly regarding management's strategic disclosure practices, remains an open question.

·References

[1] Ferreira JJ, Fernandes CI, Ferreira FA. To be or not to be digital, that is the question: Firm innovation and performance. Journal of Business research. 2019; 101: 583-590.

https://doi.org/10.1016/j.jbusres.2018.11.013

[30] Lee J, Park J. The impact of audit committee financial expertise on management discussion and analysis (MD&A) tone. European Accounting Review. 2019; 28(1): 129-150. https://doi.org/10.1080/09638180.2018.1447387

[48] Downes L, Nunes PF. Big-bang disruption. Harvard Business Review Press; 2014.

[49] Westerman G, Bonnet D, McAfee A. Leading digital: Turning technology into business transformation. Harvard Business Press; 2014.

[50] Kraus S, Jones P, Kailer N, Weinmann A, Chaparro-Banegas N, Roig-Tierno N. Digital transformation: An overview of the current state of the art of research. Sage Open. 2021; 11(3): 21582440211047576. https://doi.org/10.1177/21582440211047576

[51] Gong C, Ribiere V. Developing a unified definition of digital transformation. Technovation. 2021; 102: 102217. https://doi.org/10.1016/j.technovation.2020.102217

[52] Vial, G. Understanding digital transformation: A review and a research agenda. Journal of Strategic Information Systems. 2019; 28(2): 118-144.

https://doi.org/10.1016/j.jsis.2019.01.003

[53] Mendoza L, Ivan R. Post-truth, agnotology and instrumentality on the Internet. Andamios. 2022; 18(46): 143-169. https://doi.org/10.29092/uacm.v18i46.841

[54] Sun Q, Jiang J, Wang, Z, Liao B. Research on enterprise digital transformation disclosure based on hypocrisy theory. Journal of Intelligent & Fuzzy Systems. 2023; 44(5): 8119-8132. https://doi.org/10.3233/JIFS-224092

[55] Qu C, Liu HY. Does platform competition always improve the efficiency of information transmission? A case study of China’s search engine market in Chinese. Economic Research Journal. 2019; 54(08): 120-135.

https://link.cnki.net/urlid/11.1081.F.20190819.1737.016

[56] Chen Y, Ng J, Yang X. Talk less, learn more: Strategic disclosure in response to managerial learning from the options market. Journal of Accounting Research. 2021; 59(5): 1609-1649. https://doi.org/10.1111/1475-679X.12368

[57] Zhao T, Yan N, Ji L. Digital transformation, life cycle and internal control effectiveness: Evidence from China. Finance Research Letters. 2023; 58: 104223.

https://doi.org/10.1016/j.frl.2023.104223

[58] Chen W, Zhang L, Jiang P, Meng F, Sun Q. Can digital transformation improve the information environment of the capital market? Evidence from the analysts' prediction behaviour. Accounting & Finance. 2022; 62(2): 2543-2578.

https://doi.org/10.1111/acfi.12873

[59] Ren L, Liu J, Hao Q. How digital transformation affects the cost of equity capital: the role of information disclosure quality and stock liquidity. Industrial and Corporate Change. 2024; 33(5): 1098-122. https://doi.org/10.1093/icc/dtad053

[60] He Y, Li J, Ren Y. Digital transformation and corporate ESG information disclosure herd effect. Finance Research Letters. 2024; 65: 105557.

https://doi.org/10.1016/j.frl.2024.105557

[61] Yang GQ, Wang HS, Yue ZY, Li G. Customer enterprises’ digital transformation, suppliers’ carbon emission reduction and carbon information disclosure accommodation in Chinese. China Industrial Economics. 2024; (08): 99-117.

https://doi.org/10.19581/j.cnki.ciejournal.2024.08.005

[62] Cao YN, Zhao ZY, Sun WL. Do Managers manipulate digital transformation disclosure? Evidence from companies with negative performance aspiration gap in Chinese. Business and Management Journal. 2023; 45(06): 177-192.

https://doi.org/10.19616/j.cnki.bmj.2023.06.010.

[63] Ma LF, Song JN, Wang B. The Value Effect of Information Disclosure in Digital Transformation——Evidence from Conceptual Hype. Research on Economics and Management. 2023; 44(08): 17-37.

https://doi.org/10.13502/j.cnki.issn1000-7636.2023.08.002.

(4) Discussion of Findings: Although the results are presented clearly, the discussion could benefit from a deeper analysis of why certain control variables, such as 'Lev,' 'Indep,' and 'Soe,' have significant effects on abnormal tone. This would provide more context and depth to the findings.

**Thanks for your constructive suggestions. This paper does lack a detailed discussion of the control variables. In response to this review comment, we add the analysis of the result coefficients of the control variables in 4.2.1, as well as find existing studies to verify the results.**

In terms of the control variables, Lev, Roa, Indep, Soe, Analyst and Salary are significantly positively associated with Abtone, while Btm, Inst and HHI are significantly and negatively related to Abtone. Specifically: (1) A higher debt ratio suggests that the company faces relatively high financial risks and requires additional funds to ease debt pressures. (2) A higher return on total assets signifies robust current profitability, making managers more confident to invest in risky projects [89], which consequently increases the need for additional funds. (3) Firms with more preoccupied independent directors have declining firm valuation and operating performance and exhibit weaker accounting quality [90]. (4) Executives in state-owned enterprises hold dual roles as both "professional managers" and "government officials." They benefit not only from salary incentives but also from opportunities for political advancement. The implicit incentives for political advancement lead SOE executives to focus on building and preserving both their personal and corporate images, thereby intensifying their motivation for tone manipulation in annual reports [91]. (5) With higher compensation, management develops a strong opportunistic drive to defend their existing high wages and justify future raises. This drive to rationalize their compensation affects how the company discloses information [92,93]. In these situations, managers may manipulate tone, and this aligns with findings from prior research. Notably, both Analyst and HHI are proxies for external oversight, yet the regression coefficient for Analyst is positive. This finding aligns with [94,95]. This is because companies, to ease the increased pressure from more analysts' oversight, tend to disclose more exaggerated tones.

Other, (1) A high market-to-book ratio suggests significant investment value and growth potential, naturally drawing numerous potential investors [38]. (2) Institutional investors, in contrast to individual investors, possess higher expertise and market sensitivity, allowing them to more readily detect opportunistic management behaviors [96]. (3) Industry competition acts as an external governance mechanism, restricting opportunistic managerial behaviors [45]. In these scenarios, managers tend to reduce tone manipulation, aligning with findings from existing studies.

·References

[38] DellaVigna S, Pollet JM. Investor inattention and Friday earnings announcements. The journal of finance. 2019; 64(2): 709-749. https://doi.org/10.1111/j.1540-6261.2009.01447.x

[45] Harford J. Corporate cash reserves and acquisitions. The journal of finance. 1999; 54(6): 1969-97. https://doi.org/10.1111/0022-1082.00179

[89] Hundley G, Jacobson CK, Park SH. Effects of profitability and liquidity on R&D intensity: Japanese and US companies compared. Academy of Management Journal. 1996; 39(6): 1659-1674. https://doi.org/10.5465/257073

[90] Masulis RW, Zhan EJ. How valuable are independent directors? Evidence from external distractions. Journal of Financial Economics. 2019; 132(3): 226-256.

https://doi.org/10.1016/j.jfineco.2018.02.014

[91] Zhou J, Yuan DL, Ma YF. Does MD&A tone influence enterprises' fulfillment of social responsibility? From the perspective of Information Increment and Impression Management in Chinese. Journal of Management. 2021; 34(06): 88-107.

https://doi.org/10.19808/j.cnki.41-1408/F.2021.0046.

[92] Bebchuk LA, Fried JM. Executive compensation as an agency problem. Journal of Economic Perspectives. 2003; 17:71~92.

https://doi.org/10.1257/089533003769204362

[93] Cheng XS, Liu JM, Chen JH. Talent signaling or compensation justification: Excess executive compensation and information disclosure on strategy in Chinese. Journal of Financial Research. 2015; (12): 146-161.

[94] Du S, Yu K. Do corporate social responsibility reports convey value relevant information? Evidence from report readability and tone. Journal of Business Ethics. 2021; 172(2): 253-274. https://doi.org/10.1007/s10551-020-04496-3

[95] Yang X, Bao J, Zhang K. Environmental, social and governance (ESG) performance and abnormal positive tone. Sustainability Accounting, Management and Policy Journal. 2024; Forthcoming. https://doi.org/10.1108/SAMPJ-01-2024-0045

[96] Edmans A, Manso G. Governance through trading and intervention: A theory of multiple blockholders. The Review of Financial Studies. 2011; 24(7): 2395-428.

https://doi.org/10.1093/rfs/hhq145

**In addition, we also read the whole paper and correct the mistakes in grammar and punctuation, as well as the layout of some tables, such as Table 6. Thanks for the careful corrections on the details of this paper. These suggestions greatly improve the overall quality of this paper.**

# Reviewer #2:

1. Introduction

①“The Management Discussion and Analysis (MD&A) section, …” Compared to the rest of the Introduction, it suggested to make this paragraph a little bit "simpler"? Readers are required to receive a lot of information in few rows.

②“By employing signaling theory…” It could be critical to assume that readers easily understand the meaning of "signaling theory" and "impression management". Some reference could support the comprehension of the sentence concept.

③“It provides new perspectives …” This paragraph could benefit from some example, so that readers can better figure out the applicative implications at this early stage of the paper.

④Again, the "impression management" concept should be a bit introduced with a reference to previous studies (e.g. by Erving Goffman). Despite, it is widely diffused in the literature, a broader audience of the paper could benefit from this deepening.

⑤The end of the Introduction paragraph could help readers to have a short and clear picture of the following chapters of the paper.

**Thanks for your valuable and constructive suggestions. Your suggestions greatly help me to make the introduction more intuitive and logical. In response to this review comment, ①we modified the content of the paragraph “The Management Discussion and Analysis (MD&A) section, …” to remove the semantic fuzziness and make the sentence more intuitive, ②we add descriptions of "signaling theory" and "impression management" to help readers understand better, ③we add references to related studies in the second section of the research contribution to help readers understand better, ④we add references (Erving Goffman) to the "impression management" related content in the introduction, ⑤ we revise the last paragraph of introduction based on the subsequent structural arrangement of the article.**

①The Management Discussion and Analysis (MD&A) section serves as a primary source for conveying qualitative information in a company's annual report. It is characterized by its predictive and narrative nature, allowing for subjective and flexible information disclosure. It not only addresses investors' needs for information on business conditions, development plans, and future risks [17,18], but also serves the critical role of helping management to communicate clearly with investors [19,20].

②④This is called impression management. Impression management refers to the process where people are often concerned, both consciously and unconsciously, about how others evaluate them, and they subconsciously desire to be viewed positively. During interactions, individuals actively manage and construct their positive image to influence others' perceptions. This can lead to recognition and the establishment of a positive reputation, aiding in self-fulfillment and economic benefits [13]. Viewing through the lens of information manipulation, management is motivated to craft a positive image by exaggerating digital transformation benefits. This approach helps guide public views on the company's development, diminish negative evaluations, and enhance attention and financial support from social groups.

Signal theory posits that signals are transmitted from those with an information advantage to those at a disadvantage [14]. This theory in management explains how companies use signaling to address information asymmetry in exchanges. Companies, holding an informational advantage, disclose business and financial details to the public. This helps alleviate information asymmetry and diminish adverse selection [15,16].

③Research has investigated the impact of various factors on strategic information disclosure. These factors include a company’s financial situation [23], the level of competition in the product market [24], corporate transactions [25,26], expected future performance [27] and so on.

⑤The structure design of this paper is as follows: Section 1 is an introduction, outlines the current development status of enterprises during the digital transformation exploration period and examines the relationship between digital transformation and management's strategic information disclosure. Section 2 presents the literature review and hypotheses development, highlighting the contributions and shortcomings of relevant research. And it delves into the impact mechanism of digital transformation on management's tone manipulation and puts forward hypotheses. Section 3 is research design, including data, variable constructions, model design and research samples. Section 4 analyzes empirical regression results and conducts robustness tests. In Section 5, further analysis is conducted on the motivations and mechanism analysis behind management's tone manipulation in digitally transforming enterprises. Finally, Sections 6 summarize the main work, contributions, and limitations of this study.

·References

[13] Goffman E. The presentation of self in everyday life. 1959. Garden City, NY; 2002.

[14] Spence M. Job market signaling. Uncertainty in economics. Academic Press; 1978.

[15] Ross SA. The determination of financial structure: The incentive signaling approach. The Bell Journal of Economics. 1977; 8(1):23-40. https://doi.org/10.2307/3003485

[16] Spence M. Signaling in retrospect and the informational structure of markets. American Economic Review. 2002; 92(3): 434-459. https://doi.org/10.1257/00028280260136200

[17] Cole CJ, Jones CL. The usefulness of MD&A disclosures in the retail industry. Journal of Accounting, Auditing & Finance. 2004; 19(4): 361-388.

https://doi.org/10.1177/0148558X0401900401

[18] Bochkay K, Levine CB. Using MD&A to improve earnings forecasts. Journal of Accounting, Auditing & Finance. 2019; 34(3): 458-482.

https://doi.org/10.1177/0148558X17722919

[19] Davis AK, Piger JM, Sedor LM. Beyond the numbers: Measuring the information content of earnings press release language. Contemporary Accounting Research. 2012; 29(3): 845-868.

https://doi.org/10.1111/j.1911-3846.2011.01130.x

[20] Loughran T, McDonald B. Regulation and financial disclosure: The impact of plain English. Journal of Regulatory Economics. 2014; 45: 94-113.

https://doi.org/10.1007/s11149-013-9236-5

[23] Jung MJ, Naughton JP, Tahoun A, Wang C. Do firms strategically disseminate? Evidence from corporate use of social media. The Accounting Review. 2018; 93(4): 225-252. https://doi.org/10.2308/accr-51906

[24] Allee KD, Do C, Sterin M. Product market competition, disclosure framing and casting in earnings conference calls. Journal of Accounting and Economics. 2021; 72(1): 101405. https://doi.org/10.1016/j.jacceco.2021.101405

[25] Ertimur Y, Sletten E, Sunder J. Large shareholders and disclosure strategies: Evidence from IPO lockup expirations. Journal of Accounting and Economics. 2014; 58(1): 79-95. https://doi.org/10.1016/j.jacceco.2014.06.002

[26] Huang X, Teoh SH, Zhang Y. Tone management. The accounting review. 2014; 89(3): 1083-1113. https://doi.org/10.2308/accr-50684

[27] Graham JR, Harvey CR, Rajgopal S. The economic implications of corporate financial reporting. Journal of accounting and economics. 2005; 40(1-3): 3-73.

https://doi.org/10.1016/j.jacceco.2005.01.002

2.1. Literature review of tone manipulation

①Readers could need more information about "information game theory" and they could benefit from a clearer explanation about how it has been used in this interpretation of the management disclosure strategy.

②As above, the impression management process should be explained a little bit and cited to make its use clear in the research of this research.

③“However, Internet platforms may also facilitate …” The overall idea is clear, however some examples could benefit readers to figure out the applicative scenarios.

**Thanks for your valuable and constructive suggestions. In response to this review comment, ①we revise passages in the literature review to help readers better understand information game theory, ②we add the relevant content of impression management in the introduction section to help readers understand better, ③we illustrate some practical cases about internet platforms and information disclosure to enrich the literature review.**

① The asymmetric information game theory suggests that agents with an informational advantage engage in strategic behavior to optimize their interests through exploiting information asymmetry [41]. Managers manipulate tone for impression management to gain stock market profits, improve bond credit ratings, and stabilize their reputation and position [26,42,43]. The higher the degree of tone manipulation, the more likely the company is to engage in earnings management or use misleading information to achieve higher credit ratings [39,40,43]. These actions not only disregard the principle of accounting prudence but also increase the risk of stock price crashes [32,44]. Meanwhile, scholars explore the governance effects of management tone manipulation through various lenses, such as product market [24], industry competition [45], capital markets and legal environments [46], audit committee characteristics [30], and CEO characteristics [47].

② As shown in the second question response in the "1. Introduction".

③ For example, companies can promote and sell products through social media platforms like Weibo, Facebook, and TikTok. Xiaomi represents a successful example of using social media to disrupt traditional startup models. Another example is the interactive platforms launched by the Shenzhen and Shanghai Stock Exchanges, facilitating real-time communication. These platforms provide cost-effective and efficient communication channels, particularly benefiting small and medium investors. This approach significantly enhances the speed and fairness of acquiring information.

In practice, China's securities regulators have identified instances where misleading disclosures of popular topics confuse investors, leading to stock price manipulation. For example, companies like Bright Oceans Inter-Telecom Corporation and Jiangsu Daybright Intelligent Electric Co., Ltd. were penalized by the CSRC for ambiguously disclosing digital technology applications, such as AI and big data, in reports and interactive platforms. As a result, these companies faced penalties. The companies eventually supplemented their disclosures of related risks under regulatory pressure.

·References

[24] Allee KD, Do C, Sterin M. Product market competition, disclosure framing and casting in earnings conference calls. Journal of Accounting and Economics. 2021; 72(1): 101405. https://doi.org/10.1016/j.jacceco.2021.101405

[26] Huang X, Teoh SH, Zhang Y. Tone management. The accounting review. 2014; 89(3): 1083-1113. https://doi.org/10.2308/accr-50684

[30] Lee J, Park J. The impact of audit committee financial expertise on management discussion and analysis (MD&A) tone. European Accounting Review. 2019; 28(1): 129-150. https://doi.org/10.1080/09638180.2018.1447387

[32] Davis AK, Tama‐Sweet I. Managers’ use of language across alternative disclosure outlets: earnings press releases versus MD&A. Contemporary Accounting Research. 2012; 29(3): 804-837. https://doi.org/10.1111/j.1911-3846.2011.01125.x

[39] Clarkson PM, Kao JL, Richardson GD. The voluntary inclusion of forecasts in the MD&A section of annual reports. Contemporary accounting research. 1994; 11(1): 423-450. https://doi.org/10.1111/j.1911-3846.1994.tb00450.x

[40] Baginski SP, Demers E, Kausar A, Yu YJ. Linguistic tone and the small trader. Accounting, Organizations and Society. 2018; 68: 21-37. https://doi.org/10.1016/j.aos.2018.03.005

[41] Nikolaou IE, Chymis A, Evangelinos K. Environmental information, asymmetric information, and financial markets: A game-theoretic approach. Environmental Modeling & Assessment. 2013; 18: 615-628. https://doi.org/10.1007/s10666-013-9371-5

[42] Zeng QS, Zhou B, Zhang C, Chen XY. Annual report tone and insider trading: "Consistent appearance" or "duplicity"? in Chinese. Management World. 2018; 34(09): 143-160. https://doi.org/10.19744/j.cnki.11-1235/f.2018.09.012

[43] Lin WF, Zhao ZK, Song M. The tone manipulation of management discussion and analysis and the bondmarket reaction. Management World. 2022; 38(01): 164-180.

https://doi.org/10.19744/j.cnki.11-1235/f.2022.0012

[44] D'Augusta C, DeAngelis MD. Does accounting conservatism discipline qualitative disclosure? Evidence from tone management in the MD&A. Contemporary Accounting Research. 2020; 37(4): 2287-318. https://doi.org/10.1111/1911-3846.12598

[45] Harford J. Corporate cash reserves and acquisitions. The journal of finance. 1999; 54(6): 1969-97. https://doi.org/10.1111/0022-1082.00179

[46] Webb KA, Cahan SF, Sun J. The effect of globalization and legal environment on voluntary disclosure. The International Journal of Accounting. 2008; 43(3): 219-245.

https://doi.org/10.1016/j.intacc.2008.06.001

[47] Buchholz F, Jaeschke R, Lopatta K, et al. The use of optimistic tone by narcissistic CEOs. Accounting, Auditing & Accountability Journal. 2018; 31(2): 531-562.

https://doi.org/10.1108/AAAJ-11-2015-2292

2.2. Digital Transformation and tone manipulation

①At the beginning of the paragraph, readers could benefit from a synthetic definition of the "principal-agent problem".

②Also, the "signal theory" should be a bit introduced by a little definition and some reference.

③It is suggested to use some practical examples to clarify the effects and the biases cited (e.g. anchoring bias or "ostrich effect").

④Generally, it is suggested to make sentences shorter in order to help the readers understanding of the theoretical concepts.

⑤In particular, it is suggested to cite some reference of the Attribute substitution theory (e.g. Tversky and Kahneman when they argued the systematic errors in judgment and decision).

**Thanks for your valuable and constructive suggestions. In response to this review comment, ①we add the definition of the "principal-agent problem" in the first paragraph to help readers understand better, ②our translation of "signaling theory" is flawed and inconsistent, and "signal theory" is the same as "signaling theory", so we read the whole paper to unify the expression of signaling theory, as well as add relevant definitions and references in the introduction section,** **③we add an example description of anchoring bias and ostrich effect, ④we modified some intricate and long sentences, ⑤we cite reference of the attribute substitution theory.**

①From a micro perspective, principal-agent problems can occur between shareholders and management, shareholders and creditors, and large and small shareholders. It occurs when an agent exploits information asymmetry and conflicts of interest to harm the principal's interests. The principal, being at an informational disadvantage, is unable to fully monitor the agent [65].

②There are four point in text, revised from signal theory to signaling theory. About the changes to the content, as shown in the second question response in the "1. Introduction".

③Individual investors, when processing information, are susceptible to the information cocoon effect and the ostrich effect. And the heterogeneity of investor sentiment may make them respond differently. For instance, when expectations for future returns surpass concerns about transformation risks, investor optimism increases, potentially resulting in overconfidence. Investors with anchoring bias might overlook crucial information needed for future considerations, while those with representativeness bias may focus exclusively on information they see as representative. As a result, such biases can lead to impulsive decision-making. Conversely, if concerns about transformation risks surpass the anticipated returns, similar biases may result.

④To help readers read better, we read through the whole paper and modified some complex long sentences to make the expression more concise and direct.

⑤Attribute substitution theory suggests that individuals tend to use more readily available heuristic attributes to replace more complex and less accessible target attributes [77].

·References

[65] Jensen MC, Meckling WH. Theory of the firm: Managerial behavior, agency costs and ownership structure. Corporate governance; 2019.

[77] Kahneman D, Frederick S. Representativeness revisited: Attribute substitution in intuitive judgment. Heuristics and biases: The psychology of intuitive judgment. 2002; 49-81. https://doi.org/10.1017/CBO9780511808098.004

5.3 Mechanism analysis

When quoting different statements that come from previous studies and researches, it is suggested to always also cite the source.

Many "deductions" about managers' biases and overconfidence are common sense and reasonable, but in this context it is necessary to report from which studies these theories are taken or to which data of this study they are correlated.

**Thanks for your valuable and constructive suggestions. Our references in 5.3 section are indeed insufficient. In response to this review comment, we add references to help readers to have a clearer understanding of the existing research.**

Research on overconfidence within the academic community has gradually expanded from its early roots in psychology to include economics, management, and other fields [107,108]. Social psychology views overconfidence as an individual's overly optimistic, irrational judgment about oneself or situations. It is primarily manifested in two ways: optimistic psychological expectations or overestimating the likelihood of favorable outcomes; and overestimation of one's abilities, meaning an excessive confidence in one's judgment's effectiveness and reliability without personal bias [109]. Overconfidence, a cognitive bias in decision-making, is prevalent among corporate managers [110], epitomizing managers' irrational behavior. According to the overconfidence hypothesis and stewardship theory, cognitive biases lead overconfident managers to maintain an optimistic view of the market and the company's prospects [111]. They often overestimate their management skills, underestimate potential risks, and struggle to respond rationally to negative management feedback [112].

Previous studies have shown that past experiences and external environments are the two main sources of management's overconfidence. On the one hand, corporate management, as decision-makers, possesses significant control over resources, extensive professional knowledge, social reputation, and a history of success. These factors can lead managers to overestimate their decision-making capabilities [113]. Additionally, digital transformation represents a strategic overhaul that leverages a company's full capabilities and potential [1]. The decision-making involved in digital transformation further reinforces managers' confidence in their decision-making abilities, potentially leading to overconfidence. On the other hand, favorable evaluations and positive feedback from the external environment will increase the degree of management's overconfidence [114]. Under the digital economy's influence, investors have high expectations for the novel concept of digitalization. Consequently, this optimism towards digital transformation makes it an effective strategy for companies to garner market attention and improve market feedback [115]. The positive external reaction further amplifies the degree of management's overconfidence.

At the same time, an individual's cognitive biases not only produce overconfidence but also affect individual behavior. For example, management's overconfidence can affect the accounting conservatism of its information disclosure [116], thereby affecting the quality of information disclosure. First, managers with overconfidence tend to have overly optimistic views on the company's future, motivating them to send positive signals to investors, as suggested by signal transmission theory [107]. They manipulate the tone to display overly optimistic expectations beyond the current situation, attracting more investors' attention. Secondly, the process of digital transformation in enterprises requires continuous and substantial capital investment [117]. The overly optimistic psychology of management may lead them to participate in some inefficient resource allocation projects. Based on information asymmetry theory, management may hide negative news and embellish information with an overly optimistic tone to divert investors' attention. Finally, driven by self-interest, management strives to build a high-reputation image for benefits such as position promotion and justifying on-the-job consumption [118]. They might exaggerate the impact of digital transformation, leveraging optimistic tones to engage investors' emotions and acknowledge management's efforts in running the company.

·References

[1] Ferreira JJ, Fernandes CI, Ferreira FA. To be or not to be digital, that is the question: Firm innovation and performance. Journal of Business research. 2019; 101: 583-590. https://doi.org/10.1016/j.jbusres.2018.11.013

[107] Roll R. The hubris hypothesis of corporate takeovers. The Joumal of Business. 1986; 59 (2): 197-216. https://www.jstor.org/stable/2353017

[108] Malmendier U, Tate G. CEO overconfidence and corporate investment. The journal of finance. 2005; 60(6): 2661-2700.

https://doi.org/10.1111/j.1540-6261.2005.00813.x

[109] Griffin DW, Varey CA. Towards a consensus on overconfidence. Organizational Behavior and Human Decision Processes. 1996; 65(3): 227-231.

https://doi.org/10.1006/obhd.1996.0023

[110] Camerer C, Lovallo D. Overconfidence and excess entry: An experimental approach. American economic review. 1999; 89(1): 306-318.

https://doi.org/10.1257/aer.89.1.306

[111] Tian JJ, Lau CM. Board composition, leadership structure and performance in Chinese shareholding companies. Asia Pacific Journal of Management. 2001; 18: 245-263. https://doi.org/10.1023/A:1010628209918

[112] Taylor SE, Brown JD. Positive illusions and well-being revisited: separating fact from fiction. Psychological Bulletin. 1994; 116(1): 21-27.

https://doi.org/10.1037/0033-2909.116.1.21

[113] Alicke MD, Klotz ML, Breitenbecher DL, Yurak J, Vredenburg DS. Personal contact, individuation, and the better-than-average effect. Journal of personality and social psychology. 1995; 68(5): 804. https://doi.org/10.1037/0022-3514.68.5.804

[114] Mahajan J. The overconfidence effect in marketing management predictions. Journal of Marketing Research. 1992; 29(3): 329-342.

https://doi.org/10.1177/002224379202900304

[115] Li X, Pu H, Zhang M. Does corporate digital transformation affect investor trading behaviour? – A perspective based on investor divergence. Technology Analysis & Strategic Management. 2024; 24: 1-4.

https://doi.org/10.1080/09537325.2024.2344076

[116] Ahmed AS, Duellman S. Managerial overconfidence and accounting conservatism. Journal of accounting research. 2013; 51(1); 1-30.

https://doi.org/10.1111/j.1475-679X.2012.00467.x

[117] Zhai H, Yang F, Gao F, Sindakis S, Showkat G. Digital transformation and over-investment: Exploring the role of rational decision-making and resource surplus in the knowledge economy. Journal of the Knowledge Economy. 2023; 8: 1-32.

https://doi.org/10.1007/s13132-023-01569-2

[118] Bebbington J, Larrinaga C, Moneva JM. Corporate social reporting and reputation risk management. Accounting, Auditing & Accountability Journal. 2008; 21(3): 337-361. https://doi.org/10.1108/09513570810863932

6. Summary and conclusion

The conclusion paragraph is well structured. The numbered list helps a lot in reading the well-synthesized concepts. Point 6, unlike the first five points in the conclusions, however, seems more hypothetical than a real result of the research (it seems more like an interpretation of the authors, albeit sensible). The implications also appear very clear and targeted, useful for practical applications in politics and economics.

It is suggested to divide the conclusions paragraph into three areas: the first as now with the six numbered points, then a paragraph of "implications" and a final one of "limitations and future research" (the latter with a clear point for "Different types of digital transformation" and a final point for "Strategic use of digital transformation as an optimistic signal"). In this way, the paragraph could be even more schematic and therefore easier to read.

**Thanks for your valuable and constructive suggestions. In response to this review comment, we modify the Point 6 conclusion and rearrange the structure of the conclusion paragraph.**

Based on impression management, signal transmission, and attribute substitution theories, this study focuses on the "Management Discussion and Analysis" sections from the annual reports of Chinese A-share listed companies between 2012 and 2021. Examining the impact of digital transformation on management's tone manipulation behavior, the main research work and conclusions of this study are as follows: (1) Groups were divided based on digital transformation status to examine differences in MD&A tone manipulation by management. Findings reveal that companies, whether they have implemented digital transformation strategies, tend to portray their operations and prospects optimistically in MD&A reports. Additionally, a significant difference was observed: digitally transformed companies exhibit a higher and more pronounced optimism in their MD&A reports than their non-transformed counterparts. Consequently, transformed companies are more motivated to manage MD&A tone for specific objectives, compared to non-transformed ones. (2) In the digital economy's exploration phase, corporate digital transformation notably encouraged management's tone manipulation. Specifically, leveraging signaling theory, management is motivated to amplify digital transformation successes and obscure its challenges through tone manipulation, aiming to elicit positive market reactions to their stock price. Investors find it challenging to verify the accuracy of disclosed information, presenting an opportunity for management to manipulate information. Conversely, using attribute substitution theory, management employs "tone" to divert attention from reduced risk disclosures, preventing the dissemination of valuable, innovation-related information that competitors could exploit. (3) The more optimistic the media sentiment is, the greater its impact on how management manipulates tone during digital transformation. Media reports lacking objectivity and neutrality lead to biased sentiment, undermining their roles in information mediation and external oversight. Positive reports, misaligned with the reality of digital transformation enterprises, not only divert investors' focus from operational risks to optimistic views but also mask the overly optimistic text information steered by management's tone manipulation. (4) Companies with limited financial flexibility show a stronger correlation between digital transformation and the tendency of management to manipulate communication tone. In uncertain markets and competitive environments, the cost benefits of financial flexibility help ease the funding pressures faced by firms undergoing digital transformation. Consequently, firms with less financial flexibility undergoing digital transformation might be prone to encouraging short-sighted managerial actions, such as using tone manipulation to blur negative information and emit positive signals for specific goals. (5) Firms with less risk information disclosure show a stronger link between digital transformation and the manipulation of management's communication tone. Firms reducing risk disclosure might use a more optimistic tone in their texts to convey the disclosed information, thereby influencing investors towards decisions that benefit the firm. (6) Managerial overconfidence partially mediates the relationship between digital transformation and tone manipulation. Implementing a digital transformation strategy can boost managerial overconfidence, subsequently impacting their behaviors. One manifestation of this is tone manipulation. The objective is to steer investors toward decisions that benefit management.

This paper has some implications for the government, regulators and investors. (1) Policymakers can develop targeted policies based on successful experiences to address enterprises' financial challenges and key obstacles in digital transformation. This approach facilitates the transformation process and swiftly harnesses the competitive edge digitalization offers. (2) Regulators should focus on the form and content of information disclosure, setting clear standards for substantive content. By evaluating disclosure quality from various angles and limiting tone manipulation, they can aid enterprises in reducing disclosure costs and enhancing proactive risk information disclosure without revealing proprietary details. Simultaneously, enhancing oversight of listed companies' disclosures and increasing the costs of strategic disclosures can mitigate capital market information asymmetry and elevate disclosure quality. (3) Investors should carefully assess the optimistic tone in MD&A, comparing financial data and textual information to gauge tone anomalies and avoid being misled by strategic disclosures. They should also critically evaluate media report objectivity by comparing different sources. Furthermore, investors should focus on negative and risk information, enhancing their financial literacy through academic reports and lectures to reduce the risk of being misled by strategic disclosures.

This study also has several limitations, with future research opportunities in these main areas: (1) Different digital transformation types, such as process, service, and organizational, focus on varied aspects, potentially affecting information disclosure differently. Incorporating digital transformation types into the research framework for a comprehensive impact analysis represents a future research direction. (2) The paper examines digital transformation's influence on MD&A text tone. Exploring whether "digital transformation" as an optimistic signal, is exploited in strategic disclosures warrants further research. (3) Although this study maintains an adequate sample size, its findings are limited in generalizability as it focuses solely on Chinese A-share listed companies. Countries and markets differ significantly in their information disclosure regulations and stages of digital transformation. Future research should broaden its scope to thoroughly investigate the specific circumstances of various nations concerning information disclosure regulation and digital transformation. Comparative analyses and in-depth investigations are needed on how digital transformation influences managerial strategic information disclosure.

# Reviewer #3:

(1) The structure outlined in the final paragraph of the introduction does not fully align with the content of the subsequent chapters. It is recommended that the author revise this section to better reflect the actual structure of the paper.

**Thanks for your delicacy, there is indeed a flaw in the last paragraph of the introduction. In response to this review comment, we revise this paragraph based on the subsequent structural arrangement of the article.**

The structure design of this paper is as follows: Section 1 is an introduction, outlines the current development status of enterprises during the digital transformation exploration period and examines the relationship between digital transformation and management's strategic information disclosure. Section 2 presents the literature review and hypotheses development, highlighting the contributions and shortcomings of relevant research. And it delves into the impact mechanism of digital transformation on management's tone manipulation and puts forward hypotheses. Section 3 is research design, including data, variable constructions, model design and research samples. Section 4 analyzes empirical regression results and conducts robustness tests. In Section 5, further analysis is conducted on the motivations and mechanism analysis behind management's tone manipulation in digitally transforming enterprises. Finally, Sections 6 summarize the main work, contributions, and limitations of this study.

(2) In the literature review section, focusing solely on existing research from the perspective of motivation may come across as monotonous. It could be beneficial to further organize the literature content by also exploring its economic consequences. This would provide a more comprehensive understanding of the topic and potentially uncover new insights that could enhance the overall quality of the review.

**Thanks for your constructive suggestions. In response to this review comment, we supplement and revise the literature review section. We further sort out and elucidate the relevant literature in this field to enhance the section's richness. The paragraph added in the study is as follows:**

The asymmetric information game theory suggests that agents with an informational advantage engage in strategic behavior to optimize their interests through exploiting information asymmetry [41]. Managers manipulate tone for impression management to gain stock market profits, improve bond credit ratings, and stabilize their reputation and position [26,42,43]. The higher the degree of tone manipulation, the more likely the company is to engage in earnings management or use misleading information to achieve higher credit ratings [39,40,43]. These actions not only disregard the principle of accounting prudence but also increase the risk of stock price crashes [32,44]. Meanwhile, scholars explore the governance effects of management tone manipulation through various lenses, such as product market [24], industry competition [45], capital markets and legal environments [46], audit committee characteristics [30], and CEO characteristics [47].

·References

[24] Allee KD, Do C, Sterin M. Product market competition, disclosure framing and casting in earnings conference calls. Journal of Accounting and Economics. 2021; 72(1): 101405. https://doi.org/10.1016/j.jacceco.2021.101405

[26] Huang X, Teoh SH, Zhang Y. Tone management. The accounting review. 2014; 89(3): 1083-1113. https://doi.org/10.2308/accr-50684

[30] Lee J, Park J. The impact of audit committee financial expertise on management discussion and analysis (MD&A) tone. European Accounting Review. 2019; 28(1): 129-150. https://doi.org/10.1080/09638180.2018.1447387

[32] Davis AK, Tama‐Sweet I. Managers’ use of language across alternative disclosure outlets: earnings press releases versus MD&A. Contemporary Accounting Research. 2012; 29(3): 804-837. https://doi.org/10.1111/j.1911-3846.2011.01125.x

[39] Clarkson PM, Kao JL, Richardson GD. The voluntary inclusion of forecasts in the MD&A section of annual reports. Contemporary accounting research. 1994; 11(1): 423-450. https://doi.org/10.1111/j.1911-3846.1994.tb00450.x

[40] Baginski SP, Demers E, Kausar A, Yu YJ. Linguistic tone and the small trader. Accounting, Organizations and Society. 2018; 68: 21-37. https://doi.org/10.1016/j.aos.2018.03.005

[41] Nikolaou IE, Chymis A, Evangelinos K. Environmental information, asymmetric information, and financial markets: A game-theoretic approach. Environmental Modeling & Assessment. 2013; 18: 615-628. https://doi.org/10.1007/s10666-013-9371-5

[42] Zeng QS, Zhou B, Zhang C, Chen XY. Annual report tone and insider trading: "Consistent appearance" or "duplicity"? in Chinese. Management World. 2018; 34(09): 143-160. https://doi.org/10.19744/j.cnki.11-1235/f.2018.09.012

[43] Lin WF, Zhao ZK, Song M. The tone manipulation of management discussion and analysis and the bondmarket reaction. Management World. 2022; 38(01): 164-180.

https://doi.org/10.19744/j.cnki.11-1235/f.2022.0012

[44] D'Augusta C, DeAngelis MD. Does accounting conservatism discipline qualitative disclosure? Evidence from tone management in the MD&A. Contemporary Accounting Research. 2020; 37(4): 2287-318. https://doi.org/10.1111/1911-3846.12598

[45] Harford J. Corporate cash reserves and acquisitions. The journal of finance. 1999; 54(6): 1969-97. https://doi.org/10.1111/0022-1082.00179

[46] Webb KA, Cahan SF, Sun J. The effect of globalization and legal environment on voluntary disclosure. The International Journal of Accounting. 2008; 43(3): 219-245.

https://doi.org/10.1016/j.intacc.2008.06.001

[47] Buchholz F, Jaeschke R, Lopatta K, et al. The use of optimistic tone by narcissistic CEOs. Accounting, Auditing & Accountability Journal. 2018; 31(2): 531-562.

https://doi.org/10.1108/AAAJ-11-2015-2292

(3) In the hypotheses development section, the logic behind "Innovation, being the core of digital transformation, means that risk information disclosure often involves product research and development, technological innovation, and other related information" is not entirely clear. It is suggested that the author reconsider and revise this statement for improved clarity.

**Thanks for your constructive suggestions. There is indeed ambiguity here due to translation errors. In response to this review comment, we rearrange the translation of this sentence to express its correct meaning.**

Innovation is a fundamental aspect of digital transformation. Therefore, risk disclosures associated with digital transformation frequently include details on product development and technological innovation.

(4) Although it is a common measure, the authors are advised to specify the source of the digital transformation measure used in the main regression and to provide a list of the keywords used in the textual analysis in the appendix.

**Thanks for your constructive suggestions. In response to this review comment, we supplement the references of digital transformation measurement in 3.2.1, and list the keywords required by the text analysis method in Appendix I.**

Referring to Qi et al. (2020) [84], this study's main regression analysis quantifies enterprise digital transformation by examining the proportion of digital assets in listed companies. Specifically, the notes to the financial statements in the annual report of the enterprise disclose the details of the intangible assets. We identify keywords related to digital technology, such as "software", "network", "client", "management system" and "smart platform". These items are classified as "digital intangible assets". We add up the number of digital technology intangibles for each company in each year and then calculate their proportion of the company's intangibles for the current year. This proportion is the proxy variable of enterprise digital transformation.

Additionally, referring to Wu et al. (2021) [85], we adopt the frequency of digital transformation keywords in the annual reports of listed companies as a proxy measure of enterprise digital transformation during the robustness testing. Specifically, through text analysis, we identify feature words from five categories: artificial intelligence, blockchain, cloud computing, big data, and digital technology applications (Details in Appendix 1). Using Python 3.7.0, we extract keywords from each company's annual report and count the frequency of these keyword occurrences per company each year. Finally, we calculate the logarithm of the frequency plus one, which serves as a proxy indicator for the enterprise digital transformation.

·References

[84] Qi HJ, Cao XQ, Liu YX. The influence of digital economy on corporate governance: Analyzed from information asymmetry and irrational behavior perspective in Chinese. Reform. 2020; (04): 50-64.

[85] Wu F, Hu HZ, Lin HY, Ren XY. Enterprise Digital Transformation and Capital Market Performance: Empirical Evidence from Stock Liquidity in Chinese. Management World. 2021; 37(07): 130-144+10. https://doi.org/10.19744/j.cnki.11-1235/f.2021.0097

·Appendix 1: Keywords of Enterprise Digital Transformation

| **Category** | **Keywords** |
| --- | --- |
| Artificial Intelligence | Artificial Intelligence (AI), Business Intelligence (BI), Image Understanding, Investment Decision Support System, Intelligent Data Analysis, Intelligent Robot, Machine Learning (ML), Deep Learning (DL), Semantic Search, Biometric Technology, Facial Recognition, Speech Recognition, Identity Verification, Autonomous Driving, Natural Language Processing (NLP). |
| Blockchain | Blockchain, Digital Currency, Distributed Computing, Differential Privacy Technology, Smart Financial Contract |
| Big Data Technology | Big Data, Data Mining, Text Mining, Data Visualization, Heterogeneous Data, Credit Reporting, Augmented Reality (AR), Mixed Reality (MR), Virtual Reality (VR). |
| Cloud Computing | Cloud Computing, Stream Computing, Graph Computing, In-Memory Computing, Secure Multi-Party Computation (SMC), Neuromorphic Computing, Green Computing, Cognitive Computing, Converged Architecture, Hundred-Million-Level Concurrency, Exabyte-Level Storage (EB-Level Storage), Internet of Things (IoT). |
| Digital Technology  Applications | Mobile Internet, Industrial Internet, Mobile Connectivity, Internet Healthcare, E-commerce, Mobile Payment, Third-Party Payment, Near Field Communication Payment (NFC Payment), Smart Energy, Business-to-Business (B2B), Business-to-Consumer (B2C), Consumer-to-Business (C2B), Consumer-to-Consumer (C2C), Online-to-Offline (O2O), Online Connection, Smart Wearables, Smart Agriculture, Intelligent Transportation, Smart Healthcare, Intelligent Customer Service, Smart Home, Robo-Advisory, Smart Culture and Tourism, Smart Environmental Protection, Smart Grid, Intelligent Marketing, Digital Marketing, Unmanned Retail, Internet Finance, Digital Finance, Financial Technology (Fintech), Quantitative Finance, Open Banking |
